# Towards High-Temperature MEMS: Two-Step Annealing Suppressed Recrystallization in Thin Multilayer Pt-Rh/Zr Films

**DOI:** 10.3390/mi14112003

**Published:** 2023-10-28

**Authors:** Georgii A. Pleshakov, Ivan A. Kalinin, Alexey V. Ivanov, Ilya V. Roslyakov, Igor V. Yaminsky, Kirill S. Napolskii

**Affiliations:** 1 Department of Materials Science, Lomonosov Moscow State University, 1, Bld. 73 Leninskie Gory, Moscow 119991, Russiaikalinin@inorg.chem.msu.ru (I.A.K.); ivanov_alexey13@mail.ru (A.V.I.);; 2 Department of Chemistry, Lomonosov Moscow State University, 1, Bld. 3 Leninskie Gory, Moscow 119991, Russia; 3 Skoltech Center for Energy Science and Technology, Skolkovo Institute of Science and Technology, 3 Nobel Street, Moscow 121205, Russia; 4 Kurnakov Institute of General and Inorganic Chemistry, Russian Academy of Sciences, 31 Leninskii Avenue, Moscow 119071, Russia; 5 Department of Physics, Lomonosov Moscow State University, 1, Bld. 2 Leninskie Gory, Moscow 119991, Russia

**Keywords:** thin film, platinum–rhodium alloy, zirconium dioxide, two-step annealing, recrystallization suppression, high-temperature stability, magnetron sputtering, anodic aluminium oxide

## Abstract

Platinum-based thin films are widely used to create microelectronic devices operating at temperatures above 500 °C. One of the most effective ways to increase the high-temperature stability of platinum-based films involves incorporating refractory metal oxides (e.g., ZrO_2_, HfO_2_). In such structures, refractory oxide is located along the metal grain boundaries and hinders the mobility of Pt atoms. However, the effect of annealing conditions on the morphology and functional properties of such multiphase systems is rarely studied. Here, we show that the two-step annealing of 250-nm-thick Pt-Rh/Zr multilayer films instead of the widely used isothermal annealing leads to a more uniform film morphology without voids and hillocks. The composition and morphology of as-deposited and annealed films were investigated using X-ray diffraction and scanning electron microscopy, combined with energy-dispersive X-ray spectroscopy. At the first annealing step at 450 °C, zirconium oxidation was observed. The second high-temperature annealing at 800–1000 °C resulted in the recrystallization of the Pt-Rh alloy. In comparison to the one-step annealing of Pt-Rh and Pt-Rh/Zr films, after two-step annealing, the metal phase in the Pt-Rh/Zr films has a smaller grain size and a less pronounced texture in the <111> direction, manifesting enhanced high-temperature stability. After two-step annealing at 450/900 °C, the Pt-Rh/Zr thin film possessed a grain size of 60 ± 27 nm and a resistivity of 17 × 10^−6^ Ω·m. The proposed annealing protocol can be used to create thin-film MEMS devices for operation at elevated temperatures, e.g., microheater-based gas sensors.

## 1. Introduction

Metal thin films are extensively used in micro- and nanotechnology for the creation of various planar devices operating at elevated temperatures, e.g., fuel cells [1,2], anemometers [3,4], thermocouples [5], microheaters for infrared emitters [6], and catalytic and semiconductor gas sensors [7,8,9]. In these devices, platinum-based thin films generally play a key role in contact pads and heating elements. Pt is the most frequently used material for high-temperature applications due to its high melting point (1768 °C), one of the highest resistivities among noble metals (0.11 × 10^−6^ Ω·m at 25 °C); high-temperature coefficient of resistance (TCR, 3.9 × 10^−3^ 1/°C), stable over a wide temperature range (25–800 °C for the bulk material); and excellent chemical stability [10,11,12,13,14]. The challenge of the microheater technology is the achievement of long-term operational stability of thin-film devices at high temperatures. For instance, noticeable grain growth in platinum films with a thickness of 250 nm occurred at 400 °C [15,16,17]. Microstructural changes in thin films inevitably lead to unstable electrical characteristics, such as resistivity and TCR, which are crucial for the reliability of thin-film devices.

Several approaches have been proposed for the mitigation of delamination and recrystallization of noble metal thin films at high temperatures: (1) the deposition of bilayer structures with an adhesion layer (Ti, Ta, Cr, Zr) between the substrate and the conductive layer [17,18,19,20,21,22,23,24] or the use of an oxidizing atmosphere during the initial stage of the noble metal sputtering [25,26], (2) the sputtering of a protective dielectric layer (Si, Al_2_O_3_, Si_3_N_4_, TiO_2_, SiAlON, SiO_2_ + Si_3_N_4_) onto the top surface of metal layer [16,24,27,28,29,30], (3) alloying platinum with metals that have higher melting point (Ir, Rh) [24,31,32,33,34], and (4) the incorporation of refractory oxides (ZrO_2_, HfO_2_, Nb_2_O_3_, Y_2_O_3_, RuO_2_), acting as anchors to stabilize the grain boundaries of the metal phase [24,33,35,36,37,38]. All these approaches require recrystallization annealing at temperatures exceeding the operating temperature range of thin-film devices [17,39]. For this purpose, thin films are heated at a rate of 2–10 °C/min to the target temperature (600–1000 °C) and held at this temperature for a period of several minutes to dozens of hours [18,24,33,37]. Indicators of successful recrystallization are (i) the high electrical conductivity of the film, (ii) the absence of large defects (voids and hillocks), and (iii) narrow grain size distribution.

Currently, rhodium is one of the most widely used alloying additives for platinum [24,33]. Rhodium increases the strength characteristics of platinum at high temperatures and raises its melting point. Therefore, instead of pure platinum, platinum–rhodium alloys are often used as the material for the microheaters. It has been shown that a Pt-11 wt. % Rh alloy (or Pt_81_Rh_19_ in atomic percentages) possesses a higher thermal stability and a lower recrystallization rate in comparison to pure platinum [34].

The thermal stability of Pt_81_Rh_19_ thin films has been enhanced significantly via the incorporation of ZrO_2_ and HfO_2_ layers into film bulk [35,36]. Noticeable changes in the morphology and electrical characteristics were found only after annealing at temperatures above 800 °C. The presence of refractory oxides along the grain boundaries hindered recrystallization by anchoring the grain boundaries. It is worth noting that the resistivity values for thin films containing 10–25 at.% Zr did not differ significantly after annealing at temperatures of 750–1000 °C.

The use of porous anodic aluminium oxide (AAO) as a substrate for metal thin films offers several advantages over Si-based technology [40]. Firstly, the porous structure of AAO provides high adhesion of metal films due to a partial metal deposition into AAO pores during the film sputtering. Secondly, the thermal expansion coefficients of AAO and Pt are closely matched, reducing the risk of mechanical stresses and, as a consequence, the delamination of the metal from the substrate during heating/cooling cycles [41,42]. Thirdly, micromachining techniques (e.g., optical and electron-beam lithography) allow one to achieve high-aspect-ratio porous AAO structures with high spatial resolution due to etchant penetration into the vertically aligned pores [43]. Furthermore, AAO possesses high mechanical strength and low thermal conductivity [42,44,45], making it suitable as substrate for stable metal thin films at elevated temperatures.

Here, we present a comparative study of the annealing behaviour of Pt_81_Rh_19_ and multilayer Pt_81_Rh_19_/Zr thin films deposited onto porous AAO substrates. A two-step annealing protocol for the simultaneous formation of ZrO_2_ stabilizing agent and recrystallization of Pt_81_Rh_19_ phase was proposed, and its influence on the high-temperature stability of the thin films under study was evaluated. The morphology, crystal structure, and functional properties of the samples after annealing under different conditions were investigated. The proposed two-step annealing protocol allows us to obtain electrically conductive thin films with stable uniform morphology without hillocks and voids at temperatures of up to 1000 °C.

## 2. Materials and Methods

Porous AAO substrates were obtained via aluminium anodizing. Prior to anodizing, aluminium foils (99.999%, thickness of 500 μm) were electrochemically polished at an anodic current density of 0.63 A/cm^2^ in an aqueous solution of 1.9 M CrO_3_ and 15 M H_3_PO_4_ at 80 °C to reduce metal surface roughness. The anodizing was carried out in 0.3 M oxalic acid electrolyte in a two-electrode electrochemical cell with a volume of 5 L. The electrolyte temperature was kept constant (1.0 ± 0.5 °C) using a Petite Fleur (Huber, Edison, NJ, USA) thermostat. A titanium ring was used as a cathode. The voltage between electrodes was controlled via an N8740A (Agilent, Santa Clara, CA, USA) DC power supply. The anodizing voltage was swept at a rate of 0.5 V/s to an operating value of 100 V and then maintained throughout the entire process. The anodizing process was stopped when the charge density reached 53.1 C/cm^2^ (corresponds to the AAO thickness of 30 μm). The remained aluminium was selectively dissolved in a solution of bromine (10 vol.%) in methanol. Finally, the AAO porous films were washed with methanol and dried in air.

Metal thin films were deposited onto the top side of porous AAO substrates (opposite to the AAO side with a barrier layer) using a Q300TD Plus (Quorum Technologies, Lewis, UK) dual-target magnetron sputtering system. A sputtering chamber was pumped down to a residual pressure of 1 × 10^−4^ mbar and then filled with Ar up to a working pressure of 1 × 10^−2^ mbar. The sputtering currents were 150 mA and 60 mA for Zr and Pt_81_Rh_19_ alloy, respectively. The target to sample distance was 42 mm. The thickness of the deposits was monitored using a quartz microbalance sensor. Ten bilayers, each consisting of a 9-nm-thick Zr layer and a 16-nm-thick Pt_81_Rh_19_ layer, were deposited in the same vacuum cycle. Thus, the total thickness of multilayer metal film was about 250 nm. Pt_81_Rh_19_ film with a thickness of about 250 nm was deposited under the same conditions as a reference sample. Hereafter, metal films made of Pt_81_Rh_19_ alloy are denoted as Pt-Rh, whereas the multilayer metal structures are denoted as Pt-Rh/Zr.

The recrystallization annealing of Pt-Rh and Pt-Rh/Zr films on AAO substrates was performed in a muffle furnace L5/12 (Nabertherm, Lilienthal, Germany) in air atmosphere. A mechanical load of 4 g/cm^2^ was applied to the samples to prevent the curling of the AAO substrate during the annealing process. One- and two-step annealing approaches were used. The one-step program included heating with a constant rate of 2 °C/min to the temperatures of 800, 900, and 1000 °C, followed by a dwell time of 12 h at a target temperature. The two-step program consisted of the low-temperature step (heating with a rate of 2 °C/min to 450 °C and dwelling for 4 h) and the second high-temperature step (heating with a rate of 2 °C/min up to 800, 900, and 1000 °C and dwelling for 12 h).

The films’ morphologies were investigated via scanning electron microscopy (SEM) using Supra 50 VP (Leo, Jena, Germany) and NVision 40 (Carl Zeiss, Jena, Germany) scanning electron microscopes. The grain size distributions were determined via the statistical analysis of the SEM images using ImageJ software (v.1.50i) [46]. The elemental analysis of the metal films was performed via energy dispersive X-ray spectroscopy (EDX) using an X-Max 80 (Oxford Instruments, Abingdon, UK) detector installed in the Supra 50 VP (Leo) scanning electron microscope. The phase composition was characterized by X-ray diffraction (XRD) analysis using an ADVANCE D8 (Bruker, Billerica, MA, USA) diffractometer with Bragg−Brentano geometry and Cu Kα radiation (λ = 1.5418 Å). XRD patterns were recorded in the 25–50° 2θ range with a 0.02° step and an acquisition time of 2.5 s per step. The electrical resistivity of thin films was measured via a two-probe method [47] using a Keithley 2701 multimeter (Cleveland, OH, USA). For this purpose, 400-nm-thick gold contact pads were sputtered using a Q150T ES (Quorum Technologies) sputtering system. The contact resistance was less than 8% of the measured resistance of the thin films under study.

## 3. Results and Discussion

**Figure 1:**
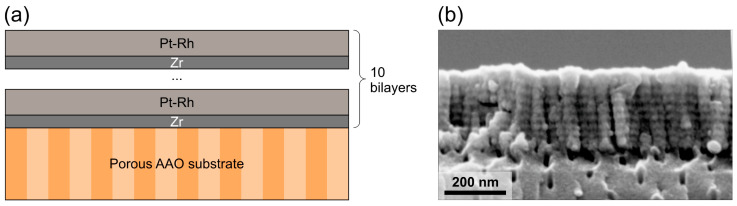
As-deposited multilayer Pt-Rh/Zr film on the porous AAO substrate: schematic cross-section of the structure under study (**a**); cross-sectional SEM image (**b**).

The structure of the as-deposited Pt-Rh/Zr multilayer film on the porous AAO substrate is shown in Figure 1 (see the schematic representation of the structure in panel (a) and the cross-sectional SEM image in panel (b)). The thickness of the metal film is 251 ± 6 nm. All the films possessed a columnar microstructure (Figure 1b) typical of magnetron sputtered Pt-based films [48,49,50].

**Figure 2:**
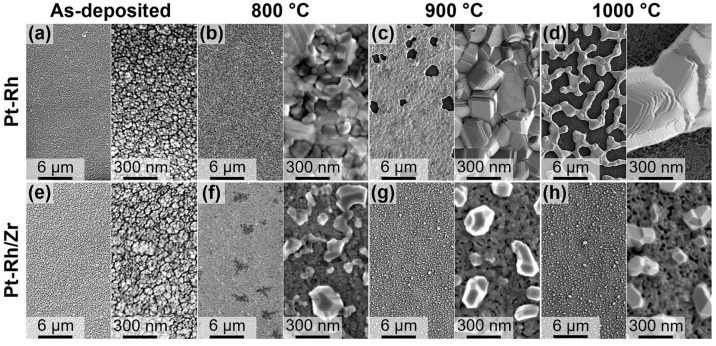
Top-view SEM images of metal films after magnetron sputtering and one-step annealing at different temperatures for 12 h. Pt-Rh films: as-deposited (**a**), 800 °C (**b**), 900 °C (**c**), and 1000 °C (**d**); Pt-Rh/Zr films: as-deposited (**e**), 800 °C (**f**), 900 °C (**g**), and 1000 °C (**h**).

Furthermore, according to the SEM data, the average grain size of as-deposited films was 15 ± 7 nm and 22 ± 5 nm for Pt-Rh and Pt-Rh/Zr, respectively (Figure 2a,e). One-step annealing at different temperatures for 12 h resulted in recrystallization accompanied by particle coarsening (Figure 2). As can be seen, the annealing of the Pt-Rh films at 800 °C led to grain growth up to 136 ± 48 nm (Figure 2b). Elevating the annealing temperature to 900 °C induced a further increase in grain size to 496 ± 120 nm, but at the same time, the formation of voids in the metal film was observed (Figure 2c). It is worth noting that the structural integrity of the conductive layer still preserved. On the contrary, after annealing at 1000 °C, the Pt-Rh films lost their structural integrity (Figure 2d). The growth of the grains occurred inhomogeneously, which manifested in the high dispersion of the grain size (1496 ± 736 nm).

**Figure 3:**
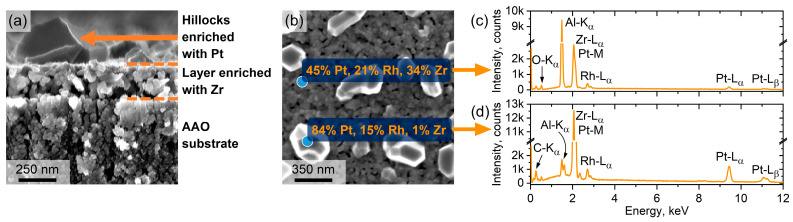
SEM images of the Pt-Rh/Zr film after one-step annealing at 900 °C for 12 h: cross-section (**a**) and top-view (**b**). The panel shows the elemental composition in atomic percentages according to EDX analysis. Corresponding EDX spectra of a layer enriched with Zr (**c**) and a hillock enriched with Pt (**d**).

In the case of the Pt-Rh/Zr films, one-step annealing resulted in completely different morphology (Figure 2f–h). The hillocks were formed on the surface of continuous fine-grained bottom layer (Figure 3a). Moreover, the bottom layer contained a significant number of pores (see dark areas in Figure 2f–h). The simultaneous presence of the pores and hillocks most likely indicates the partial diffusion of metal atoms from the bulk of the film to its surface. The results of the statistical analysis of grain size distribution are summarised in Table 1.

According to the EDX data, the as-deposited Pt-Rh film consisted of 81.0 ± 0.4 at.% Pt and 19.5 ± 1.4 at.% Rh, whereas the Pt-Rh/Zr multilayer film comprised of 61 ± 6 at.% Pt, 14 ± 1 at.% Rh, and 25 ± 2 at.% Zr. The EDX analysis revealed an enhanced concentration of platinum (84 at.% Pt, 15 at.% Rh, and 1 at.% Zr) in the hillocks formed during one-step annealing on the surface of the Pt-Rh/Zr films (Figure 3b,d). On the contrary, the fine-grained bottom layer was enriched with zirconium (45 at.% Pt, 21 at.% Rh, and 34 at.% Zr), in comparison to the composition of the as-deposited Pt-Rh/Zr film (Figure 3c). Thus, if one-step isothermal annealing is applied, the zirconium layers in the film bulk do not suppress the recrystallization of the Pt-Rh alloy. Since the annealing stages of Zr and Pt-Rh are performed simultaneously, zirconium does not completely oxidize before the recrystallization of Pt-Rh takes place.

**Figure 4:**
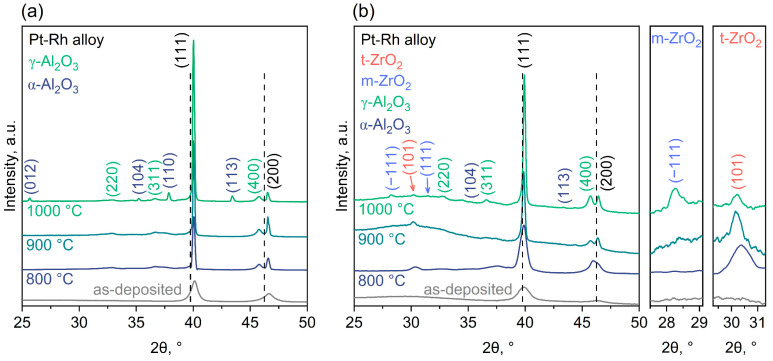
XRD patterns of Pt-Rh (**a**) and Pt-Rh/Zr (**b**) films after one-step annealing at different temperatures. The right parts of (**b**) demonstrate enlarged 2θ intervals for (−111) reflection of monoclinic ZrO_2_ (m-ZrO_2_) and (101) peak of tetragonal ZrO_2_ (t-ZrO_2_). The dash lines indicate the positions of the (111) and (200) Pt peaks according to ICDD PDF-2 [4-802].

XRD data for the as-deposited Pt-Rh and Pt-Rh/Zr films and the samples subjected to one-step annealing at different temperatures are shown in Figure 4. The most intense peaks at 2θ = 40.0° and 46.6° in the XRD patterns of the samples with Pt-Rh film are attributed to the (111) and (200) Pt-Rh reflections, respectively (Figure 4a). The rest of the reflections are from the AAO substrate. After annealing at 800 °C, amorphous AAO transformed into γ-Al_2_O_3_ (ICDD PDF-2 [10-0425]). The increase in temperature to 900 °C did not noticeably change the phase composition. After annealing at 1000 °C, the crystallization of α-Al_2_O_3_ (ICDD PDF-2 [10-0173]) occurred. The observed phase transformations of AAO are in a good agreement with those in previous reports [51,52,53].

There are several features of the XRD patterns of Pt-Rh films. For the as-deposited film (111), the (200) peaks of Pt-Rh are low-intensive and broad, since the film consists of small grains (Table 1) with low crystallinity. The recrystallization process occurring during annealing manifests itself in the increase in intensity and the decrease in full width at the half maximum (FWHM) of the (111) Pt-Rh peak. These parameters change monotonically with annealing temperature, which corresponds to a more complete recrystallization at a high temperature. Metallic platinum (ICDD PDF-2 [4-802]) and rhodium (ICDD PDF-2 [5-685]) have a face-centered cubic lattice and crystallize in the Fm−3m space group. Moreover, Rh has smaller cell parameter (*a* = 3.8081 Å) in comparison to Pt (*a* = 3.9231 Å). Therefore, the positions of the peaks in the experimental XRD pattern are shifted to higher 2θ values relative to the positions of the corresponding peaks for Pt. We should also stress a pronounced texture in the <111> direction for Pt-Rh films. The texture coefficient *T*_hkl_ was calculated using the Harris formula, as described elsewhere [54]. In the case of a non-texturized sample, it is equal to one for all the peaks in the XRD pattern. For the Pt-Rh films under study, an increase in the texture coefficient *T*_111_ from 0.93 (for as-deposited film) to 1.80 (after annealing at 1000 °C) was observed.

The described features for the position, intensity, and FWHM of the diffraction peaks are also observed in the XRD patterns of the Pt-Rh/Zr thin films (Figure 4b). It is worth noting that after the annealing of multilayer Pt-Rh/Zr films at 1000 °C, the texture coefficient lies in the range *T*_111_ = 1.35–1.55, which proves the suppression of Pt-Rh recrystallization using Zr additives. Furthermore, after annealing at 800 °C and 900 °C, peaks corresponding to tetragonal zirconium dioxide (t-ZrO_2_, ICDD PDF-2 [42-1164]) can be clearly seen. For the bulk materials, the monoclinic phase (m-ZrO_2_, ICDD PDF-2 [37-1484]) is stable below 1170 °C, whereas the tetragonal phase of ZrO_2_ is thermodynamically stable only in the range from 1170 to 2370 °C [55]. In thin films, size and microstrain effects significantly affect phase stability. In particular, the stabilization of the t-ZrO_2_ in the thin films at room temperature has previously been proven [56,57,58]. At a certain critical annealing temperature, the zirconia grains become significantly coarser as a result of recrystallization, and the size and microstrain effects become weaker. A partial transformation of t-ZrO_2_ into a monoclinic phase was observed after annealing at 1000 °C (Figure 4b). A similar effect was previously reported in [35], where, at slightly higher annealing temperature (1050 °C), a complete transformation of t-ZrO_2_ into m-ZrO_2_ was observed.

**Figure 5:**
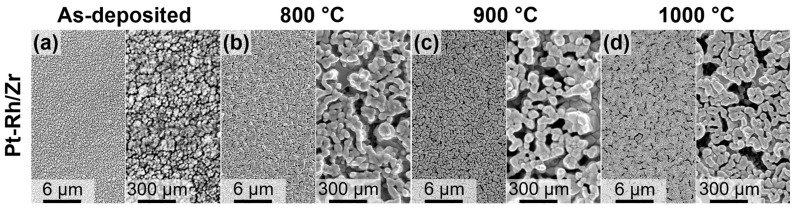
SEM images of the as-deposited Pt-Rh/Zr film (**a**) and Pt-Rh/Zr films after two-step annealing at different temperatures in the second step: (**b**) 800 °C, (**c**) 900 °C, and (**d**) 1000 °C.

Taking into account that refractory metal oxides, but not the metal additives, can suppress recrystallization, two-step annealing program was proposed. At the first step, Pt-Rh/Zr films were annealed in air at 450 °C for 4 h. These conditions were chosen based on the results of [59], as they were sufficient for the oxidation of 9-nm-thick Zr layers in the 250 nm Pt-Rh/Zr thin films. We should stress that the Pt-Rh recrystallization rate at 450 °C is negligible [34]. At the second step, Pt-Rh/Zr films were subjected to recrystallization annealing in air at 800, 900, and 1000 °C for 12 h. The SEM images of the Pt-Rh/Zr films after two-step annealing are shown in Figure 5. The films have similar fine grain morphology without hillocks for all the annealing temperatures. The average grain size of the Pt-Rh/Zr films after two-step annealing is much smaller than that after one-step annealing (Table 1). According to the EDX data, the elements were uniformly distributed over the film’s surface, and their content was found to be the same as in the as-deposited film (61 ± 4 at.% Pt, 14 ± 2 at.% Rh, and 25 ± 3 at.% Zr).

**Figure 6:**
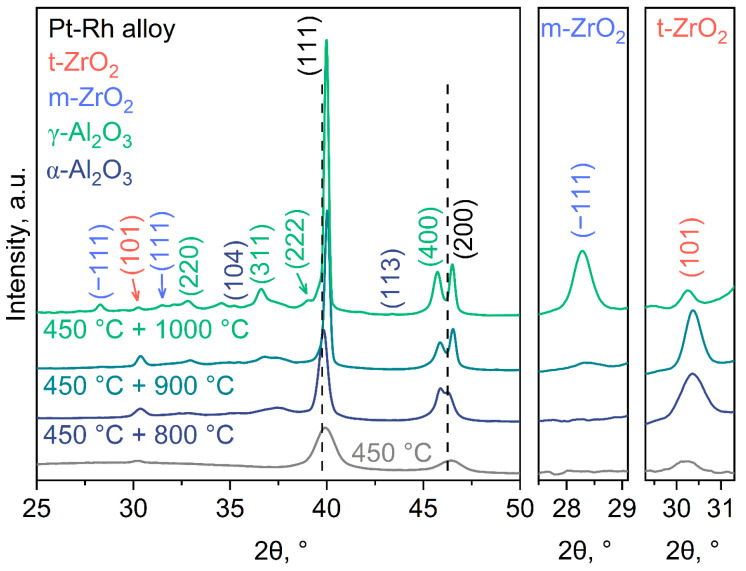
XRD patterns of Pt-Rh/Zr films after two-step annealing. The insets on the right demonstrate enlarged 2θ intervals for the (−111) reflection of monoclinic ZrO_2_ (m-ZrO_2_) and the (101) peak of tetragonal ZrO_2_ (t-ZrO_2_). The dash lines indicate the positions of the (111) and (200) Pt peaks according to ICDD PDF-2 [4-802].

In the case of Pt-Rh/Zr film annealed at 450 °C, three peaks are observed in the XRD patterns at 2θ = 30.3°, 40.0°, and 46.6° corresponding to (101) t-ZrO_2_, (111) Pt-Rh, and (200) Pt-Rh, respectively (Figure 6). This clearly indicates the occurrence of zirconium oxidation at 450 °C during the first step of the annealing. A further rise in the annealing temperature leads to the increase in the t-ZrO_2_ peak intensity, which may indicate the continuous oxidation of metallic zirconium or an improvement in the crystallinity of the t-ZrO_2_ phase. At 1000 °C, the expected partial phase transition from t-ZrO_2_ to m-ZrO_2_ was observed. Thus, the main reason for the preservation of the film microstructure is the partial oxidation of zirconium at the first annealing step, which subsequently suppresses the recrystallization of the Pt-Rh alloy.

**Figure 7:**
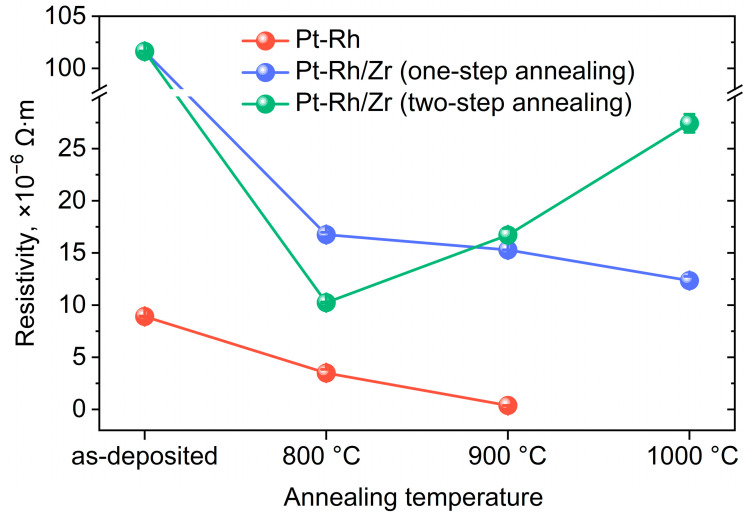
Electrical resistivity of the Pt-Rh and Pt-Rh/Zr thin films annealed at different temperatures.

The resistivity of the samples under study is shown in Figure 7. As-deposited Pt-Rh film possesses electrical resistivity of an order lower than for the as-deposited Pt-Rh/Zr multilayer structure due to the high resistivity of zirconium (0.42 × 10^−6^ Ω·m at 25 °C) [60] or its partial oxidation in air. Annealing at 800 °C for 12 h leads to a decrease in the resistivity of both Pt-Rh and Pt-Rh/Zr films owing to the grain growth and, consequently, a decrease in the electron scattering at the grain boundaries. The lowest resistivity (0.38 × 10^−6^ Ω·m) was observed for the Pt-Rh film annealed at 900 °C that is comparable to the resistivity of the bulk Pt_81_Rh_19_ (0.19 × 10^−6^ Ω·m) [61]. However, at 1000 °C, the Pt-Rh film lost structural integrity and electrical conductivity. In contrast, Pt-Rh/Zr films demonstrated the resistivity in the range 10–25 × 10^−6^ Ω·m for all the annealing temperatures.

According to the XRD, SEM, EDX, and resistivity data, the Pt-Rh/Zr multilayer thin films after two-step annealing at 450/900 °C are promising for the creation of microelectronic devices operating at high temperatures. Recrystallization under these annealing conditions is significantly suppressed and results in the thin films with low resistivity, uniform morphology, fine grain size, and the absence of hillocks and large voids. Therefore, the further evolution of the microstructure at typical operation temperatures of gas sensors of about 500 °C is improbable.

## 4. Conclusions

In summary, a series of 250-nm-thick Pt-Rh and multilayer Pt-Rh/Zr thin films were prepared on porous anodic alumina substrates using magnetron sputtering and annealed at different temperatures using one- or two-step protocols. Zirconium incorporation suppressed the recrystallization of the Pt-Rh films, which manifested themselves in the decrease in the grain size from 496 ± 120 nm to 202 ± 69 nm in the case of one-step annealing at 900 °C for 12 h. Pt-Rh films without zirconium lost conductivity after annealing under the mentioned conditions, whereas the resistivity of Pt-Rh/Zr films at room temperature was about 20 × 10^−6^ Ω·m and demonstrated weak dependence on annealing temperature from 800 to 1000 °C. However, one-step annealing of the Pt-Rh/Zr multilayer films at 800, 900, and 1000 °C for 12 h leads to an unfavorable film structure possessing platinum-enriched hillocks and a fine-grained bottom layer enriched with zirconium dioxide. The use of the two-step annealing process allows us to oxidize zirconium at 450 °C before the intensive recrystallization of the Pt-Rh alloy at 800–1000 °C. As a result, the Pt-Rh/Zr thin films after two-step annealing demonstrate a three times smaller grain size and uniform morphology compared to the ones after one-step annealing at the same temperature. In particular, the Pt-Rh/Zr thin films after two-step annealing at 450/900 °C possess a grain size of 60 ± 27 nm and a resistivity of 17 × 10^−6^ Ω·m. Thus, the separation of the processes of the refractory metal oxidation and recrystallization of a platinum-based alloy is critical for obtaining high-quality conducting films needed for microheaters and other MEMS devices operated at elevated temperatures.

## Figures and Tables

**Table 1:** The grain size of Pt-Rh and Pt-Rh/Zr thin films annealed at different temperatures for 12 h.

	Annealing Temperature	As-Deposited	800 °C	900 °C	1000 °C
Sample					
Pt-Rh (one-step annealing)		15 ± 7 nm	136 ± 48 nm	496 ± 120 nm	1496 ± 736 nm
Pt-Rh/Zr (one-step annealing; size of hillocks)		22 ± 5 nm	140 ± 89 nm	202 ± 69 nm	210 ± 65 nm
Pt-Rh/Zr (two-step annealing)			44 ± 20 nm	60 ± 27 nm	64 ± 20 nm

## Data Availability

All data that support the findings of this study are presented in the manuscript. Raw data for X-ray diffraction and scanning electron microscopy are available from the corresponding author upon reasonable request.
